# The role played by family physicians in providing health services for the sheltered homeless populations during COVID-19 lockdown in the Tshwane district

**DOI:** 10.4102/phcfm.v13i1.3060

**Published:** 2021-10-28

**Authors:** Edith N. Madela-Mntla, Wayne Renkin, Johannes F. Hugo, Paul S. Germishuys

**Affiliations:** 1 COPC Research Unit, Department of Family Medicine, Faculty of Health Sciences, University of Pretoria, Pretoria, South Africa; 2 Department of Family Medicine, Faculty of Health Sciences, University of Pretoria, Pretoria, South Africa

**Keywords:** family physicians, homeless population, homeless shelter, research report, health services, COVID-19, lockdown, Tshwane District

## Abstract

This short report describes the role that family physicians (FPs) (and family medicine registrars) played to provide care for the homeless people in shelters (both temporary and permanent) during the coronavirus disease 2019 (COVID-19) lockdown in the City of Tshwane, South Africa. The lockdown resulted in the establishment of a large number of temporary shelters. The FPs took on the task to provide comprehensive and coordinated primary care, whilst extending their activities in terms of data management, quality improvement, capacity building and research. The FPs worked in teams with other healthcare providers and contributed a unique set of skills to the process. This report demonstrates the value of responding quickly and appropriately through communication, cooperation and innovation. It also demonstrates the large number of areas in which FPs can make a difference when engaged appropriately, with the necessary support and collaboration, thus making a positive impact in the already overburdened health services.

## Background

When the South African President announced the initial lockdown at the start of the coronavirus disease 2019 (COVID-19) pandemic in South Africa (SA), he stated that ‘temporary shelters that meet the necessary hygiene standards will be identified for homeless people’.^1^ However, there was no clear strategic plan, and it was implied that provincial and local governments would be responsible for the implementation of the plan. With the historical failure in the City of Tshwane (CoT) to address homelessness, the lack of dedicated budgets for implementing the *Street Homelessness policy for the CoT*,^2^ led initially to sporadic responses by the local government, which became more coordinated during the weeks that followed.^3,4^ This failure is detailed, with the extent of the issue, in two reports– *Pathways out of Homelessness: Research Report 2015*^5^ and *Homelessness and COVID-19 in the CoT: Research report 2021*.^3^ The 2015 report resulted from a transdisciplinary research project with over 40 researchers from various disciplines and universities, as well as practitioners and people with lived experiences of being homeless. It found that there were 6244 homeless persons in the CoT, which led to the 2019 CoT Street Homelessness policy. The 2021 report details the efforts towards addressing homelessness during COVID-19 lockdown in the CoT (April–August 2020). It describes the collaboration between more than 24 organisations and institutions to develop 1800 new bed spaces, ensuring access to healthcare for the homeless. It also highlights the use of technology and effective communication.

The Department of Family Medicine (DFM) at University of Pretoria has had a strong presence in communities around the CoT for a few years already, working closely with the district healthcare services. One of its notable achievements is the formation of the Community Oriented Primary Care Research Unit (COPC RU),^6^ which was established in 2016 to create a service learning research platform. The Unit has established university-run clinics in Daspoort as well as in the informal settlements of Melusi, Zamazama (west of Pretoria) and Woodlane (east of Pretoria). These clinics aspire to offer services to the whole population that (1) are coordinated for effective, equitable quality healthcare delivery; (2) are patient-centred and delivered from the home, street, clinic or general practice to the hospital and back again; and (3) include an academic platform to support an apprenticeship model of education, workplace learning, research, monitoring and evaluation – supported by modern technology. It was against this background that the Gauteng Department of Health (GDH) and the CoT approached the DFM to assist with health interventions during the COVID-19 lockdown. It was an opportune time for the DFM to become more innovative, as Tshwane District Hospital had been converted to a COVID-19 hospital and the registrars needed alternative placement for their professional growth. The DFM rolled out health interventions to include homeless shelters and managed the coordination of healthcare for residents, with support from the GDH and other partners.

## Family medicine

The clinical specialty of Family Medicine is patient-centred, evidence based, family-focused, and problem-oriented.^7^ It emphasises the relationship with the patient and seeing the person as a whole, in the context of their family (next of kin or relevant others) and their wider community.^8^ The location of family physicians (FPs) in the African continent varies from tertiary hospitals (Nigeria) to health districts as part of district clinical specialist teams, district hospitals or in primary care (SA).^9^ We interpret these settings as care in the home, clinic or hospital and includes homeless people where the home is the street or a shelter.^10^

## Focus

This short report focuses on the role played by FPs (and family medicine registrars) in providing healthcare for the sheltered homeless people during the first 6 months (April 2020 to September 2020) of the COVID-19 lockdown^11^ in the CoT. It examines how FPs responded with agility to the unprecedented chaotic situation. Furthermore, it describes the health interventions implemented by the DFM and how the roles of the FPs extended to communication, collaboration and innovation.^12^ It also highlights FPs’ roles in capacity building, community-based care, person-centred care, continuity of care, linkage to care and multi-disciplinary teams. In providing clinical care, FPs also worked with clinical associates who are part of the Community Oriented Substance Use Programme (COSUP).^13^

The relevance of this report is that it examines the roles that can be played by FPs during public health crises, and highlights the importance of their role within community-based multidisciplinary teams.

## Context

The interventions are located geographically in the CoT, Gauteng, SA, which is within the health district of Tshwane. The timeline of the report is the first 6 months of the COVID-19 lockdown. Family physician-led multidisciplinary teams rolled out healthcare interventions in both permanent and temporary shelters (totalling 25) managed by various stakeholders, and extended these interventions to other settings, such as primary health care (PHC) clinics and buildings, used by the homeless.

## Roles played by family physicians

Table 1 shows the thematic or operational areas in which FPs played a role in providing healthcare services for sheltered homeless populations during the 2020 COVID-19 lockdown in the Tshwane district.

**TABLE 1:** Thematic or operational areas of the roles played by family physicians.

Thematic or operational area	Roles or description
Clinical care	Provision of directly observed therapy (DOT) for opioid substitution therapy Initiation and maintenance of medication for acute and chronic diseases identified. Telemedicine consultations for shelters across Tshwane
Care coordination and collaboration	Hosting multi-site daily Zoom meetings for care coordination across shelters and facilities. To cope with increasing COVID-19 positive cases from mid-July, all FPs in the district were included in the weekly Zoom meeting to coordinate district-wide efforts to contain COVID-19 and agree on clinical approaches. A project was started to deliver pre-packaged chronic medication to patients at home and all shelters to avoid big crowds at PHC clinics and promote access to care – a ‘home delivery’ service normally available only to patients with medical aid. As cases started climbing around mid-July, FPs started doing more collaboration with other healthcare services and provincial authorities in the form of organising transportation for the transfer of patients between different health facilities and health care levels, using the Mpilo app. Additional vehicles were temporarily organised through a private provider to transport staff, equipment, and patients, as part of coordination of healthcare between the different care levels: shelter, PHC clinic and hospital.
Health screening	Screening for diseases, harmful substance use, screening and testing for COVID-19, and linking positive cases to care
Technological mastery	Designing ad-hoc data collection tools Harmonisation of data across tools Learning to use new technological tools
Data collection and management	Registering and screening the homeless for diseases and substance use. Adapting patient data to the Qualtrics^14^ tool and later the Phulukisa^15^ tool for harmonisation. Collection of clinical data and supervision of data capturers for appropriate data collection. Family physicians checked and cleaned data to eliminate or correct corrupt, incomplete, incorrect and irrelevant data. A project was started to deliver pre-packaged chronic medication to patients at home and all shelters to avoid big crowds at clinics – a ‘home’ delivery service normally available only to people with medical aid. The Vula Mobile app^16^ was used to trigger referrals to other specialists. The Mpilo app^17^ was used for the organising of transport for patients.
Quality improvement	In a dilapidated building with 115 residents, a quality improvement project with final year medical students screened people for TB and HIV. (This is an ‘abandoned’ government building, and people occupied it because they have no other alternatives – therein they developed communities and systems, but there are no municipal services).
Capacity building	Visiting PHC clinics to provide and build capacity on proper use of personal protective equipment, as well as proper procedures for screening and testing for COVID-19. Providing cloth masks to clients and health talks on hand washing and social distancing. Training shelter managers on COVID-19 protocols.
Research	A number of research studies were initiated on different aspects of homelessness and health care. Recommendations were made for long-term care of homeless people from a health perspective.

FP, family physician; COVID-19, coronavirus disease 2019; TB, tuberculosis; HIV, human immunodeficiency virus; PHC, primary health care.

## Strengthening primary care and district healthcare services

The DFM, through the COPC RU’s programmes such as COSUP,^14^ has been providing healthcare services to homeless people on the street for 5 years. The establishment of new shelters and the influx of additional homeless people to the existing shelters were an opportunity to provide integrated healthcare to homeless populations, rather than expecting that they would come to healthcare facilities.

The initiative provided integrated healthcare for people in their context. Although the care was provided in crowded, noisy settings, it was an appropriate service for the residents to get a comprehensive and integrated care, tailored to their identified needs. Family physicians moved beyond their comfort zones and learnt a number of skills which were not usual for their workplace^4^ (Table 1). This disruption provided an opportunity in strengthening the district healthcare services that were stretched to the limit at that time, both by the high numbers of sick people needing care simultaneously and by an unusually high number of ‘captive’ populations needing care.

The high ‘captive’ population situation was a result of street people, normally lost to the system, gathering at the many shelters. An opportunity arose to collect data and gain deeper understanding of the health needs of homeless people. An unprecedented number of substance users were initiated on opioid substitution therapy.^18^ Moreover, others got weaned off harmful substance use, either because of not finding suitable alternatives to their usual substances or embracing lifestyle changes through other services offered.

The homeless were screened and assessed for various conditions, and initiated or reinitiated on treatment, a move that increased access to healthcare in the district for that group. Technology utilisation improved in the district health system, as FPs interacted mainly via Zoom, telemedicine and Vula Mobile application^16^ to exchange information, and had to learn fast how to compile data and use data collection tools, which was previously unfamiliar.

## Impact of the family physician-led activities

The impact of FPs’ activities in providing healthcare services to the sheltered homeless populations during the COVID-19 lockdown in Tshwane district can be considered according to six previously defined roles: care provider, consultant, clinical trainer, leader of clinical governance, champion of community-orientated primary care and capacity builder.^19^ Table 1 lists the operational areas in which FPs played a leading role and made a significant impact in healthcare services. It demonstrates how FPs went beyond their roles expected in SA’s health system. The unique contributions of FPs to the teams were the following: initiation of medication for acute and chronic diseases identified; telemedicine consultations; care coordination across shelters and facilities; development of district-wide standard clinical approaches to contain COVID-19; screening for diseases and harmful substance use, as well as screening, swabbing and linkage to care for COVID-19; collection of clinical data and training of data capturers for appropriate data collection and cleaning; using the Vula application for specialist referrals; undertaking a quality improvement project, as well as training shelter managers on COVID-19 protocols.

Table 2 illustrates the impact made by one team of FPs providing services in a particular shelter over three of the 6-month period. By caring for the homeless people outside of the primary care facilities, they also reduced the workload of these facilities. It shows the numbers attended to daily through the different alert levels. It also illustrates early detection of diseases, initiation and continuity of clinical care and linkage to health services. The pandemic peaked during alert level 3 lockdown,^11^ which started in June, whilst the DFM continued service delivery. As numbers declined from July, some residents voluntarily left the shelters whilst those remaining were gradually transferred to permanent shelters.

**TABLE 2:** Family physician activities at one homeless shelter – April to June 2020.

Time period	Total at shelter	Number increase %	Total seen	% Seen	Total unseen	% unseen	Total patients seen	Total chronic	Total acute	Follow-up patients	Total OST users	COVID screened	Total referred				Total masks given	Flu vaccines	Total HIV tests	TB screening
													Acute illness	Chronic illness	Mental Health	TB				
1 April – 15 April	350	0.0	244	69.7	106	30.30	425	38	232	155	29	341	31	2	4	3	0	0	27	3
16 April – 30 April	379	7.6	345	91.0	34	8.97	522	2	259	264	20	525	18	8	0	5	324	220	4	5
Total April	379	7.6	345	91.0	34	8.97	947	40	491	419	49	866	49	10	4	8	324	220	31	8
1 May – 15 May	382	0.8	380	99.4	2	0.52	333	26	169	119	15	333	5	17	2	0	105	0	8	0
16 May – 31 May	384	0.5	383	99.7	1	0.26	280	25	155	86	15	280	10	14	1	0	10	0	57	0
Total May	384	1.3	383	99.7	1	0.26	613	51	324	205	30	613	15	31	3	0	115	0	65	0
1 June – 15 June	389	1.3	389	100.0	0	0.00	204	23	111	67	3	191	4	5	1	1	0	0	0	1
16 June – 30 June	393	1.0	393	100.0	0	0.00	245	21	158	66	0	200	11	13	1	1	406	0	3	1
Total June	393	2.3	393	100.0	0	0.00	449	44	269	133	3	391	15	18	2	2	406	0	3	2

**Total in time peried**	**393**	**11**.**2**	**393**	**100.0**	**0**	**0.00**	**2009**	**135**	**1084**	**757**	**82**	**1870**	**79**	**59**	**8**	**10**	**845**	**220**	**99**	**10**

OST, opioid substitution therapy; HIV, human immunodeficiency virus; COVID, coronavirus disease; TB, tuberculosis.

## Conclusion

As lead clinicians in primary care, FPs can adapt to emergency situations and play a role beyond what is normally expected of them within district healthcare services in South Africa. The disruption resulting from the first 6 months of the COVID-19 lockdown in the country presented an opportunity for the FPs to go beyond their normal clinical roles by immersing themselves into additional activities, including data management, research and effective utilisation of technology. Family physicians also played an extended role in capacity building as a result of this initiative, which included training on the wearing of masks, proper hand washing, and training on COVID-19 swabbing for nurses and clinical associates. Begun and Jiang^12^ suggest that an appropriate response during the COVID-19 pandemic is to ‘emphasise communication, collaboration, and innovation’, as well as building connections, at great speed. The responses from FPs during the COVID-19 pandemic, as highlighted in this report, were therefore appropriate.
